# Multifunctional and Redundant Roles of *Borrelia burgdorferi* Outer Surface Proteins in Tissue Adhesion, Colonization, and Complement Evasion

**DOI:** 10.3389/fimmu.2016.00442

**Published:** 2016-10-21

**Authors:** Jennifer A. Caine, Jenifer Coburn

**Affiliations:** ^1^Division of Infectious Disease, Center for Infectious Disease Research, Medical College of Wisconsin, Milwaukee, WI, USA

**Keywords:** Lyme disease, adhesion, *Borrelia*, colonization, complement, pathogenicity, virulence

## Abstract

*Borrelia burgdorferi* is the causative agent of Lyme disease in the U.S., with at least 25,000 cases reported to the CDC each year. *B. burgdorferi* is thought to enter and exit the bloodstream to achieve rapid dissemination to distal tissue sites during infection. Travel through the bloodstream requires evasion of immune surveillance and pathogen clearance in the host, a process at which *B. burgdorferi* is adept. *B. burgdorferi* encodes greater than 19 adhesive outer surface proteins many of which have been found to bind to host cells or components of the extracellular matrix. Several others bind to host complement regulatory factors, *in vitro*. Production of many of these adhesive proteins is tightly regulated by environmental cues, and some have been shown to aid in vascular interactions and tissue colonization, as well as survival in the blood, *in vivo*. Recent work has described multifaceted and redundant roles of *B. burgdorferi* outer surface proteins in complement component interactions and tissue targeted adhesion and colonization, distinct from their previously identified *in vitro* binding capabilities. Recent insights into the multifunctional roles of previously well-characterized outer surface proteins such as BBK32, DbpA, CspA, and OspC have changed the way we think about the surface proteome of these organisms during the tick–mammal life cycle. With the combination of new and old *in vivo* models and *in vitro* techniques, the field has identified distinct ligand binding domains on BBK32 and DbpA that afford tissue colonization or blood survival to *B. burgdorferi*. In this review, we describe the multifunctional and redundant roles of many adhesive outer surface proteins of *B. burgdorferi* in tissue adhesion, colonization, and bloodstream survival that, together, promote the survival of *Borrelia* spp. throughout maintenance in their multi-host lifestyle.

## Introduction

*Borrelia burgdorferi*, a diderm motile spirochete bacterium, is the causative agent of Lyme disease in the U.S. Each year greater than 25,000 confirmed cases of Lyme disease are reported to the United States Centers for Disease Control and Prevention with about 96% of those cases reported from only 14 states in the Midwest and East (1). Lyme disease is also a significant health problem in parts of Europe and Asia where it is more commonly caused by *Borrelia afzelii* and *Borrelia garinii* than *B. burgdorferi*.

*Borrelia* spp. are maintained in nature in a tick–mammal life cycle. *B. burgdorferi* is carried by several species of the *Ixodes* genus of tick and is transmitted to mammals through tick saliva (2). The spirochetes are maintained in the tick midgut as the tick progresses through its life stages, but the bacteria are not passed transovarially to its offspring (2). The primary mammalian reservoir for *B. burgdorferi* is the white-footed mouse, *Peromyscus leucopus* (2). This reservoir is not known to be physically affected by the infection (3). Additional small animals and birds can also serve as reservoirs, whereas large animals and humans can be accidental hosts for tick feeding and subsequent infection.

Human infection with *Borrelia* spp. often results in a number of generic symptoms including headache, fatigue, and general malaise, and for this reason, the infection is often misdiagnosed or goes untreated. A large percentage of individuals infected with *B. burgdorferi* will display a rash, termed erythema migrans, at the site of a tick bite (4). An untreated infection with *B. burgdorferi* can result in late stage symptoms including arthritis, carditis, and neurologic issues (5–7). The CDC reported from 2001 to 2010 that 31% of confirmed Lyme disease cases presented with Lyme arthritis, 14% with neurologic symptoms, and 1% with cardiac involvement (1). The results of a late stage *Borrelia* infection vary depending on the infecting species, with *B. garinii* most often associated with neurologic symptoms and *B. afzelii* infection commonly associated with a skin rash called acrodermatitis chronica atrophicans (8–10).

## Outer Surface Proteins of *Borrelia burgdorferi*

**Figure 1:**
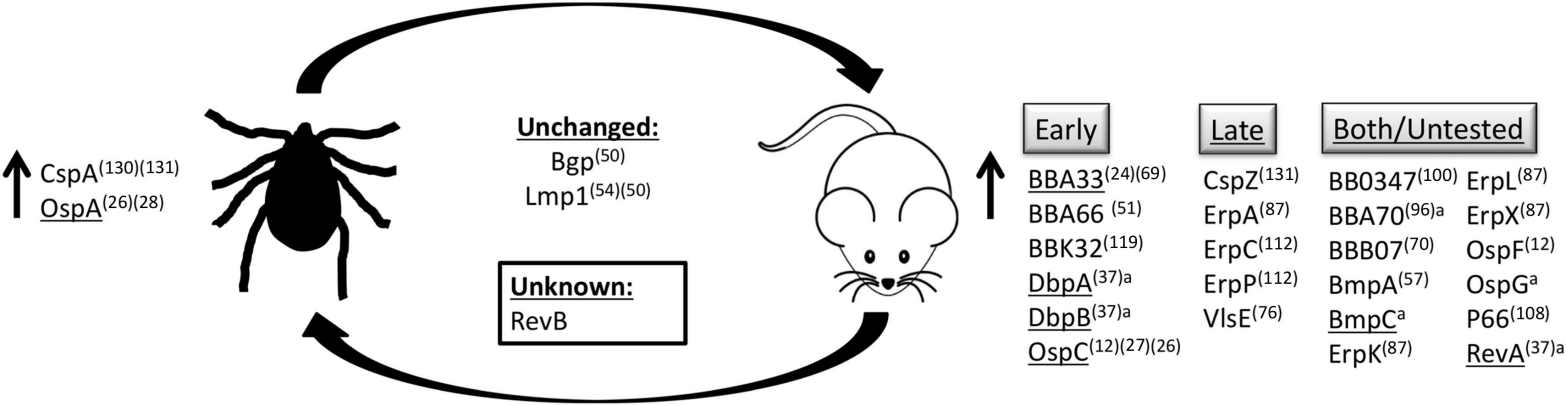
**Outer surface protein regulation**. *B. burgdorferi* senses changes in temperature, pH, and cell density, as well as unknown stimuli to modulate production of proteins on the bacterial surface. Proteins listed are upregulated in their respective environments, in the tick vector or the mammalian host, or are produced at similar levels in both environments. Proteins produced during mammalian infection are grouped based on temporal expression pattern, expressed early during infection (early), during persistent infection (late), or those that have been detected both early and late during infection or have not been experimentally determined (both/untested). ^a^Based on *in vivo* qRT-PCR and microarray data (15). ^(#)^Reference. Underline indicates genes regulated by the RpoS regulon.

*Borrelia* spp. are able to exist in the tick–mammal life cycle due to their ability to adapt to the environment in which they reside. In *in vitro* studies, *Borrelia* spp. are able to respond to changes in pH and temperature of the environment, as well as cell density of spirochetes, to differentially regulate the production of many of their outer surface proteins (Figure 1) (11–14).

One way in which *B. burgdorferi* is able to respond to changes in these environmental conditions is through the RpoN–RpoS signaling system (14, 16). RpoS, RpoN, Rrp2, and BosR are considered the master regulators of virulence gene expression in *B. burgdorferi* (17–23). RpoS and Rrp2 have been shown to be required for mouse infectivity (18, 24). One example of such control is the reciprocal expression of outer surface protein A (*ospA*) and outer surface protein C (*ospC*) tightly regulated by RpoN, RpoS, and Rrp2 (19–21, 25, 26).

*Borrelia burgdorferi* produces OspA on its surface while in the unfed tick (11). Upon the uptake of blood into the midgut of the tick, *ospA* expression is maintained until transmission into the mammal when expression is decreased and *ospC* expression is increased in conjunction with many other genes that encode outer surface proteins, to aid in survival within the mammal (15, 27, 28). Interestingly, OspC production is not necessary and is, in fact, detrimental to survival of the bacteria, likely due to the high immunogenicity of the OspC protein (29). In addition to regulation of surface protein production by RpoN, RpoS, Rrp2, or BosR, *B. burgdorferi* also utilizes other mechanisms to rapidly change the epitopes available on the surface inside the mammalian host, but not within the tick (30). For example, *B. burgdorferi* encodes a variable membrane protein-like sequence (Vls) antigenic variation system that enables evasion of recognition by the host-adaptive immune system by continual recombination of silent *vls* gene segments encoding different *vlsE* sequences into the expression site (31–34).

### Outer Surface Proteins and Virulence

For years, a focus of the *Borrelia* field, as with any pathogen field, has been to identify bacterial proteins that could contribute to virulence. Due to the cumbersome nature of *Borrelia* genetics, the roles of very few proteins have been described in mammalian infection. Using traditional cloning methods, glycosaminoglycan (GAG) and fibronectin (Fn)-binding protein, BBK32, and GAG and decorin-binding proteins, DbpA and DbpB, were all identified as being important for the establishment or persistence of mammalian infection (35–44). Additionally, the Fn-binding protein, RevA, was also found to have an effect on bacterial virulence (45, 46), though the affinity of the interaction between RevA and Fn was found to be less than that of BBK32 and Fn (41). Likewise, deletion of the *ospC* gene from *B. burgdorferi* strain B31 also has a negative impact on the establishment of infection in mice (29, 38, 47). This may be due to the antiphagocytic properties of OspC on *Borrelia*, though the importance of this activity has not yet been elucidated *in vivo* (48). Recent headway has been made in identifying *Borrelia* genes involved in mammalian infection by the generation and utilization of a transposon library in *B. burgdorferi* (49). By inoculating mice with the signature-tagged transposon mutagenesis (STM) library, the list of virulence determinants of *B. burgdorferi* was expanded to include additional outer surface proteins, many with undescribed function including BBB07, Bgp, BmpC, ErpA, RevB, and VlsE (Table 1) (49, 50). Through the use of traditional cloning, it was shown that the lipoprotein, BBA66, is also required for mammalian infection (51, 52).

**Table 1:** **Adhesive outer surface proteins of *B. burgdorferi***.

Adhesin	Genetic locus^a^	*In vitro* binding	Reference	*In vivo* function	Reference
**Adhesins with a role in mammalian infection**					
Lmp1	*bb0210*	Chondroitin-6-sulfate	(53)	Not determined	(53–55)
BmpA	*bb0383*	Laminin	(56)	Joint persistence	(57)
BmpC	*bb0384*	Not determined		Not determined	(49)
Bgp	*bb0588*	Heparin, dermatan sulfate, GAGs, and aggrecan	(58–60)	Not determined	(49, 58)
P66	*bb0603*	Integrins αIIbβ3 and αvβ3	(61)	Heart and skin adhesion, dissemination, and vascular transmigration (integrin-binding domain)	(62–64)
DbpA	*bba24*	Decorin, GAGs	(65–68)	Joint colonization	(29, 35–44, 47, 49)
DbpB	*bba25*	Decorin, GAGs	(65–68)	Joint colonization	(36, 43, 44, 49)
BBA33	*bba33*	Collagen	(69)	Not determined	(69)
BBB07	*bbb07*	Integrin α3β1	(70)	Not determined	(49)
OspC	*bbb19*	Plasminogen, Salp15 (in tick saliva)	(71, 72)	Bloodstream survival	(29, 38, 47, 62)
RevB	*bbc10*	Fibronectin	(41, 45)	Not determined	(49)
VlsE	*bbf32*	Not determined		Not determined	(49, 73–76)
BBK32	*bbk32*	Fibronectin, GAGs, and complement component C1r	(77–80)	Vascular adhesion and joint colonization (GAG-binding domain)	(35, 39–42, 49, 62)
RevA	*bbm27, bbp27*	Fibronectin, laminin	(41, 45)	Heart colonization	(46, 49, 62)
ErpA (CRASP-5)	*bbp38*	Factor H, Factor H-related proteins 1, 2, and 5, and plasminogen	(81–86)	Not determined	(49, 87)
**Adhesins with no role in mammalian infection**					
OspA	*bba15*	TROSPA (in tick)	(88)	N/A	(89)
CspZ (CRASP-2)	*bbh06*	Factor H, Factor H-like 1	(82, 90)	N/A	(91)
**Adhesins with an undetermined role in mammalian infection**					
CspA (CRASP-1)	*bba68*	Factor H, Factor H-like 1, and complement components C7 and C9	(86, 92–95)	Not determined	
BBA70	*bba70*	Plasminogen	(96)	Not determined	
ErpC (CRASP-4)	*bbl39*	Factor H, Factor H-related protein 1, and plasminogen	(82, 83, 85)	Not determined	
ErpK	*bbm38*	Heparan sulfate	(97)	Not determined	
ErpP (CRASP-3)	*bbn38*	Factor H, Factor H-related proteins 1, 2, and 5, and plasminogen	(81–85, 98)	Not determined	
ErpL	*bbo39*	Heparan sulfate	(97)	Not determined	
ErpX	*bbq47*	Laminin	(85)	Not determined	
OspF	*bbr42*	Heparan sulfate	(97)	Not determined	
ErpG (OspG)	*bbs41*	Heparan sulfate, heparin	(97)	Not determined	

*N/A, not applicable*.

*^a^All gene designations are based on B. burgdorferi strain B31*.

### Ligand Binding Mediated by *Borrelia burgdorferi* Outer Surface Proteins

*Borrelia burgdorferi* is known to produce at least 19 adhesive proteins on its surface (Table 1) (35). Previous work has focused on describing the binding capability of three *B. burgdorferi* outer surface proteins to the ECM component, Fn, which is expressed by a variety of cells types and has been shown to be important in neural and vascular development (99): RevA, BBK32, and BB0347. RevA is a 17 kDa outer surface lipoprotein of *B. burgdorferi* produced within the mammalian environment, which has been shown to bind to Fn *in vitro*, though reports on the affinity of this interaction are conflicting (41, 45). Recently, it was found that production of RevA is required for the colonization of heart tissue 1 month p.i. (46). In addition, *B. burgdorferi* produces BB0347 during mammalian infection (100), which has also been shown to bind to Fn with low affinity by surface plasmon resonance (41). RevA and BB0347 were both found to have a minimal role in vascular binding in mouse flank skin *in vivo* 1-h post infection (h.p.i.) (41). Much work has been done to decipher the role of the surface protein BBK32, produced during mammalian infection, and its high affinity interactions with Fn and GAGs, which are evenly distributed throughout the ECM of all mammalian tissues (41, 77, 101, 102). Tissue distribution and function of GAGs was expertly reviewed by Jinno and Park (86). Early work with BBK32 identified distinct regions of the protein required for binding to GAGs (residues 45–68) and Fn (residues 158–182) (41, 103). In contrast to other Fn-binding adhesins on *B. burgdorferi*, the region of Fn that interacts with BBK32 has been precisely defined (41). Interactions of BBK32 on the surface of *B. burgdorferi* with GAGs and Fn are important for tethering and dragging interactions with endothelial cells of the vasculature *in vivo*, respectively (33, 39, 98). This was demonstrated by a restoration of vascular adhesion to non-adhesive mutant *B. burgdorferi* upon BBK32 production as determined by intravital microscopy in mouse flank skin (35, 41, 104). Another component of the ECM, collagen, which is a structural component of bone, tendon, and ligaments [as reviewed in Ref. (99)] also acts as a ligand for *B. burgdorferi*. BBA33, was shown to bind to collagen type VI *in vitro*, and is required for mammalian infection (69). Additionally, the basement membrane glycoprotein, laminin, found in the epithelium [as reviewed in Ref. (99)], has been shown to be a ligand for *B. burgdorferi* adhesins BmpA, ErpX, and RevA (41, 56, 105). Studies using BmpA-deficient *B. burgdorferi* showed a role for this protein in bacterial persistence in the joints of mice (57).

Adhesive surface proteins of *B. burgdorferi* have also been identified that bind to integrins, integral membrane proteins found on the surface of all nucleated mammalian cells that function to bind to several ECM components [as reviewed in Ref. (106)]. One such protein is the 66 kDa putative porin, P66, which has been shown to be important for the establishment of mammalian infection (63, 107, 108). P66 was shown to bind to β3-chain integrins and is involved in bacterial dissemination from the site of inoculation in the skin (64, 109). *B. burgdorferi* encodes another integrin-binding protein, BBB07, which is produced during mammalian infection as evidenced by the presence of a specific antibody response in the serum of infected individuals (70). This protein has been shown to have a role in signaling through integrin α3β1 to induce the production of pro-inflammatory cytokines *in vitro*, though this activity has not yet been described *in vivo* (70).

Additional outer surface proteins (Osp) have been described on *B. burgdorferi* that are involved in binding to other host proteins, including the 22 kDa outer surface protein, OspC. OspC production is induced upon bacterial entry into mammalian tissue (Figure 1) (19) and has been shown to bind plasminogen *in vitro* (71). Plasminogen is a mammalian protein important for the degradation of the ECM to facilitate cellular migration. OspE protein family members, ErpA, ErpC, and ErpP, were all found to bind to plasminogen *in vitro*, as is seen with a number of other *Borrelia* proteins (85, 96, 110–112). However, the presence or role of plasminogen binding by these proteins *in vivo* has not yet been described. A function for plasminogen binding has been recently described for the adhesive *B. burgdorferi* protein, BBA70. Through *in vitro* experiments, it was shown that BBA70 binds to plasminogen, which cleaves and inactivates complement component C5, ultimately inhibiting membrane attack complex formation (96).

Along with producing proteins that bind to Fn, integrins, and plasminogen, *Borrelia* spp. also produce proteins, which have been shown to bind to a variety of GAGs. Just as was seen with BBK32, outer surface proteins DbpA and B, produced during mammalian infection (37), have been shown to bind to decorin, heparin, dermatan sulfate, and heparan sulfate *in vitro* (66–68, 113–115). OspF-related family members, ErpG, ErpK, and ErpL, were all found to bind to heparan sulfate, in addition to plasminogen, with varying affinities as determined by a series of *in vitro* assays (97). To add a layer of complexity, DbpA from different *Borrelia* species was found to bind to dermatan sulfate with differing affinities, which may contribute to the differences seen in clinical manifestations of disease caused by *B. garinii, B. burgdorferi*, and *B. afzelii* (114).

## Tissue Colonization by *Borrelia burgdorferi*

In order to colonize tissue sites distal to the site of tick bite in the mammal, it is thought that *Borrelia* travel within the vascular system of the host. Specific tissue colonization by the spirochetes is thought to occur by targeted exit from the vasculature dictated by the binding specificity of the outer surface proteins of *Borrelia* spp. This hypothesis is plausible, as characteristics of vascular beds differ depending on the tissue they are associated with as well as the size and type of vessel (116, 117). Tissue bed-specific endothelial cell surface receptors have been documented including VCAM1 in liver, CD36 in lung and heart, L-selectin (CD62L/SELL) in the spleen, and CD133 in the skin, brain, eye, and testicular microvasculature (116). Vascular beds can also vary in their production of proteoglycans, GAGs, and ECM components [as reviewed in Ref. (118)]. The ability of *B. burgdorferi* to bind to GAGs and ECM components has been specifically associated with effects on bacterial virulence and tissue colonization for *Borrelia* outer surface proteins such as DbpA and BBK32 (36, 39, 42–44, 51, 71, 77, 113, 119).

It is possible that *B. burgdorferi* are able to interact with differentially available endothelial cell surface proteins and utilize them to preferentially bind different tissues. Endothelial cell binding by *B. burgdorferi* has been observed to occur *in vitro* through interaction with cell surface proteoglycans such as dermatan sulfate, fibronectin, and heparin (120, 121). The binding of *B. burgdorferi* to the vasculature has been observed, *in vivo*, to occur in three stages (35, 104). Using intravital microscopy techniques on mouse flank skin, Norman et al. observed both tethering and dragging interactions to occur during the first stage of *B. burgdorferi* interactions with the vasculature (35). Using a flow chamber, tethering and dragging interactions with the endothelium were confirmed to occur both at the cell surface and at endothelial cell junctions (121). Stationary interactions, which occur after tethering and dragging, may be controlled by *B. burgdorferi* integrin-binding proteins such as P66 and BBB07. After the spirochete is tightly associated with the endothelial cell junction, it is able to transmigrate into the tissue where it is presumed to colonize and replicate.

By inoculating mice with a library of filamentous phage-expressing *B. burgdorferi* N40 D10/E9 gene fragments, a number of outer surface proteins of *B. burgdorferi* that have a tropism for particular mouse tissues were identified (122). Several of the identified proteins in these experiments have been shown to bind to a small number of host proteins, as described in Table 1. This redundancy in protein function suggests a high importance for tissue tropic binding by the bacteria during a mammalian infection. The tissue targeting effects of the different outer surface proteins of *Borrelia* may be due to the presence of nutrients required for survival of the bacteria at those particular sites. The cumulative data suggest an association between *in vitro* ligand affinity and the ability to bind to particular tissues *in vivo*. For example, the GAG-binding proteins BBK32 and DbpA from *B. burgdorferi* have been shown to be tropic for joint tissue in mouse models of *Borrelia* infection (40, 114). Detailed work with BBK32 has shown the joint targeting effects of the protein to be mediated specifically by the GAG-binding domain (40). This association between ligand affinity and tissue targeting does not hold true with Fn-binding adhesins. Fn-binding proteins, BB0347 and BBK32, have been shown to preferentially bind joint tissue (40, 62), whereas the Fn-binding protein, RevA, was found to have a tropism for heart tissue *in vivo* (62). These data highlight the inherent difficulties in using *in vitro* binding data between two proteins to infer *in vivo* function of a protein. It is becoming increasingly clear that the outer surface proteome of *Borrelia* spp. during mammalian infection functions collaboratively to direct the bacteria to specific tissue sites. Further experiments using *in vivo* models will need to be performed to determine functions for each outer surface protein of Lyme disease *Borrelia*.

Interestingly, tissue tropism of *Borrelia* spp. appears not only to be determined by different bacterial proteins but also by allelic differences in a gene encoding a given protein taken from different *Borrelia* species. For example, *dbpA* gene sequences are variable between strains as well as species of *Borrelia*, and this variation not only affects ligand binding, as mentioned earlier, but also tissue tropism (114). It was shown that DbpA from *B. burgdorferi* strains B31-A3 and N40 D10/E9 is tropic for joint tissue adhesion and colonization, while DbpA from *B. afzelii* and *B. garinii* is not (114). DbpA from *B. afzelii* strain VS461 showed a tropism for skin, while DbpA from *B. garinii* strain PBr was uniquely tropic for heart tissue colonization among the *dbpA* alleles tested (75). These differences in tissue tropism of allelic gene variants from different strains and species of *Borrelia* could explain the differences observed in the symptoms of a late stage infection with *Borrelia* spp. DbpA may contribute to these symptoms as *B. burgdorferi* infection often results in arthritis symptoms, while late stage *B. afzelii* infection commonly presents with skin lesions.

## Complement Component Binding

Rapid dissemination of *B. burgdorferi* during an infection is thought to be facilitated by the spirochetes traveling in the bloodstream of the mammalian host. During this dissemination process, the bacteria are exposed to the innate immune system of the host, designed to detect and clear invading pathogens. *B. burgdorferi* is also exposed to these blood products inside the midgut of a feeding tick during a blood meal. Resistance to killing by innate immune mechanisms in the blood and host tissues is, therefore, essential for maintenance of the enzoonotic lifestyle of *B. burgdorferi*.

One of the major innate immune components in the host blood and tissues is the complement cascade. The complement cascade is a series of proteolytic cleavage events in which inactive precursors are converted into active enzymes in the host serum and tissues. These proteolytic cleavage events are activated by three distinct pathogen-recognition mechanisms. The first is termed the “classical” complement cascade, mediated by antibodies that recognize the surface of the pathogen and recruit complement component C1q molecules, which then activate the cascade of enzymatic cleavage events. Mannose-binding lectin molecules present in the serum can bind to sugar moieties on the surface of an invading pathogen, activating the complement cascade by the “lectin” pathway. A third pathway known as the “alternative” pathway activates the complement cascade by random deposition of complement component C3 molecules onto the surface of the pathogen, activating the cascade. All three of the complement pathways converge on the activation of complement component C3, ultimately resulting in formation of the membrane attack complex pore in the pathogen membrane, and pathogen lysis.

The host has adapted many different regulatory mechanisms to control the activation of this pathway to minimize harm to host tissues. One such mechanism is the C1 inhibitor protein (C1-INH), which acts on the C1s and C1r molecules of the first step of the classical pathway (123). Additionally, the host produces C4-binding protein (C4BP), which inhibits the classical and lectin pathways by binding to C4b and inducing its proteolytic cleavage and subsequent inactivation [reviewed in Ref. (124)]. The host also has a number of different mechanisms that it employs for regulating the alternative complement cascade. The host produces Factor H, Factor H-like protein 1 (FHL-1), and complement Factor H-related (CFHR) proteins, which all inhibit the alternative complement cascade by acting on C3b. Factor H can act to sterically hinder the interactions of C3b with Factor B, compete with Factor Bb for binding to C3b, and induce its proteolytic degradation and inactivation into iC3b [reviewed in Ref. (125)]. Regulation of the complement cascade can occur at the end stages of the cascade by employing factors such as clusterin (ApoJ), vitronectin, and CD59. Vitronectin is known to inhibit the binding of complement factor complex C5–C7 to the pathogen membrane, as well as inhibit C9 oligomerization (126). CD59 functions in a similar fashion to vitronectin, intercalating into the membrane attack complex, and inhibiting its polymerization. Clusterin is a complement regulatory glycoprotein associated with apolipoprotein AI, a protein component of high-density lipoprotein (HDL) cholesterol molecules (125). Clusterin inhibits insertion of the membrane attack complex into the pathogen membrane by forming attack complex aggregates away from the pathogen surface (125).

*Borrelia* spp. produce several proteins on their surfaces, which are proposed to allow them to evade clearing by the complement cascade. Many of the proteins act by recruiting complement regulatory factors to the surface of *B. burgdorferi*, such as the surface proteins CspA (86, 92–95), CspZ (82, 90, 91), ErpP (94), ErpA (81, 98), and ErpC (127–129) as determined *in vitro*. Full length CspA and ErpP were found to be required for binding to purified human Factor H and FHL-1 *in vitro* using a series of C-terminal truncation proteins in a solid phase binding assay (92). The binding of Factor H and FHL-1 by CspA on the surface of *B. burgdorferi* was confirmed by far western blot and immunofluorescence assays (95) and contributes to cleavage and inactivation of complement component C3b (93). CspA has also been shown, *in vitro*, to interact with complement components C7 and C9, at a distinct location from the site of Factor H binding on CspA (92, 93, 95). Binding of CspA to C7 and C9 inhibits assembly of the membrane attack complex at the spirochete surface when incubated in active human serum as determined by immunofluorescence microscopy, contributing to bacterial resistance to lysis by human serum proteins *in vitro* (92, 93, 95). Interestingly, *cspA* was found to be expressed only in an unfed tick and not during mammalian infection, suggesting a necessary role for complement resistance of *Borrelia* within the tick (130, 131). Similar to CspA, another outer membrane protein of *B. burgdorferi*, CD59-like protein, also binds complement component C9 as well as C8β and inhibits the insertion of the membrane attack complex *in vitro* (132).

*Borrelia burgdorferi* also produces several OspE-related protein family members including ErpA, ErpP, and ErpC, which have been shown to recruit Factor H and CFHR proteins to the bacterial surface *in vitro* (81–83). By solid phase-binding assay and immunoblot, ErpA, ErpP, and ErpC were found to bind to CFHR-1, unlike CspA and CspZ, which showed no binding to CFHR-1 in active human serum *in vitro* (82). Additionally, recombinant ErpP and ErpA were found to bind full length recombinant Factor H with a low dissociation constant, suggesting a potential role for this interaction *in vivo*, though this has not yet been established (81, 83).

*CspZ*, a gene encoding another Factor H and FHL-1-binding protein on the surface of *B. burgdorferi*, has a reciprocal expression pattern to *cspA* (90). *CspZ* was found to be expressed primarily during mammalian infection but was not found to be required for establishment of mammalian infection (91). Consistent with this result, CspA and CspZ on the surface of *B. burgdorferi* were found to interact with human Factor H and FHL-1 *in vitro*, although production of CspZ was not found to be required for survival in active human serum *in vitro* (82, 91).

Recently, BBK32 was found to interact with a component of the classical complement cascade *in vitro* (80). BBK32 was shown to bind C1r, a member of the first protein complex in the classical complement cascade, whereby inhibiting its zymogen activity and effectively blocking attack complex formation *in vitro* (80). A C-terminal portion of BBK32 (residues 206–354) outside of the Fn- and GAG-binding domains, was found to be necessary for the interaction with C1 *in vitro* (80). We would predict based on the *in vitro* binding capabilities of BBK32 to C1, that BBK32 cooperates *in vivo* with additional *Borrelia* surface proteins to ensure successful inhibition of multiple branches of the complement cascade.

Experiments have been performed to discern the role of complement in the clearance of *Borrelia* during mammalian infection using C5-deficient mice (133). Complement component C5 is a point of convergence of all three pathways of the complement cascade. The activation of C5 ultimately results in the formation of the membrane attack complex pore in the pathogen membrane. *B. burgdorferi* recovery by culture from tissues of several infected strains of mice naturally deficient in C5, including A/J, AKR/J, B10.D2/oSnJ, DBA/2J, and SWR/J, was not found to be different than recovery from infected C5 sufficient C3H/HeJ mouse tissues at 2, 4, and 12 weeks p.i. (133). The authors concluded that complement activity is not required for clearance of *B. burgdorferi* during a mouse infection. Similar results were seen in experimentally infected C3-deficient mice where complement sensitive *B. garinii* was not recovered from complement-deficient mice (134). Additionally, it was shown that recruitment of Factor H to the surface of *B. burgdorferi in vivo* is not necessary for serum resistance of the bacteria as evidenced by similarities in bacterial burdens of WT and Factor H-deficient mice (135). Given what we now know about the ability of *B. burgdorferi* to interact with several host complement regulatory factors upstream of C3 and C5 *in vitro*, one would not predict to see a difference in WT bacteria survival in these experiments.

The evasion of the alternative complement cascade is not unique to *B. burgdorferi* and has been observed in *Borrelia hermsii*, a causative agent of relapsing fever (136, 137), *Borrelia bavariensis* (138), as well as other bacterial pathogens such as *Streptococcus pyogenes* (139), *Bordetella pertussis* (140, 141), *Neisseria gonorrhoeae* (142–145), *Escherichia coli* (146–148), *Leptospira interrogans* (149), *Yersinia pseudotuberculosis* (150, 151), *Salmonella enterica* serovar Typhimurium (152), and *Moraxella catarrhalis* (153). Many of these organisms obtain resistance to host complement by coating themselves in C4-binding protein (C4BP) and Factor H, similar to *B. burgdorferi* [reviewed in Ref. (124)].

## Summary

*Borrelia* spp. are highly adept at preventing clearance by the innate immune system of the host while adhering to and colonizing host tissues. The success of this mammalian pathogen can, so far, be attributed to the presences of a few bacterial proteins produced on the pathogen surface though there are many more adhesins whose *in vivo* functions have yet to be determined. For many years, researchers in the *Borrelia* field have identified and studied outer surface proteins of *Borrelia* spp. that may contribute to the efficiency of *Borrelia* as a pathogen. The host proteins that are specifically bound by different outer surface proteins are beginning to be revealed. *Borrelia* outer surface proteins BBK32, DbpA, RevA, CspA and Erps A, C, and P, have all been shown to bind to multiple macromolecules from the host cell surface and ECM. In addition to binding to multiple host cell surface proteins, *Borrelia* proteins BBK32, CspA, and Erps A, C, and P have all been found to bind to host complement components. The multifunctional and redundant capabilities of many of the proteins on the surface of *Borrelia* provide the bacteria with a higher probability of successfully infecting the arthropod and mammalian hosts. As research of *Borrelia* outer surface proteins progresses, it becomes increasingly clear that redundancy in ECM and host protein-binding specificity acts to guarantee that the bacteria will successfully colonize host tissues while evading detection by the host immune system.

As suggested in this review, there is a strong correlation between ligand specificity of different bacterial outer surface proteins and the tissue tropism of those proteins. When examining the literature it becomes apparent that the binding affinity for host macromolecules such as GAGs by BBK32 and DbpA may be the necessary interactions required for joint tropism and colonization (40, 114). This is further evidenced by the documented species to species differences in GAG-binding capacity and joint colonization seen with the production of DbpA from *B. afzelli* and *B. garinii, Borrelia* species, which do not commonly cause arthritis in humans (114).

As discussed in this review, complement protein resistance is achieved by *Borrelia* spp. through the action of a few outer surface proteins with multiple levels of redundancy. One aspect of redundancy is the ability of multiple *Borrelia* proteins to bind to and recruit the same complement inhibitory protein. This functional redundancy ensures the successful inhibition of the complement cascade at different stages of the pathway by multiple *Borrelia* proteins. Redundancy in protein function is a common theme for both the tissue binding and complement protein recruitment aspects of *Borrelia* pathogenesis, and likely contribute largely to the pathogenic success of this bacterial genus.

## Author Contributions

JAC and JC both contributed to the writing of this manuscript.

## Conflict of Interest Statement

The authors declare that the research was conducted in the absence of any commercial or financial relationships that could be construed as a potential conflict of interest.
